# Family context as a double-edged sword for psychological distress amid the COVID-19 pandemic with the mediating effect of individual fear and the moderating effect of household income

**DOI:** 10.3389/fpubh.2023.1109446

**Published:** 2023-03-23

**Authors:** Bowen Chen, Weijie Gong, Agnes Yuen Kwan Lai, Shirley Man Man Sit, Sai Yin Ho, Nancy Xiaonan Yu, Man Ping Wang, Tai Hing Lam

**Affiliations:** ^1^Department of Social and Behavioural Sciences, City University of Hong Kong, Kowloon, Hong Kong SAR, China; ^2^Department of General Practice, Medical School, Shenzhen University, Shenzhen, China; ^3^School of Public Health, The University of Hong Kong, Pokfulam, Hong Kong SAR, China; ^4^School of Nursing, The University of Hong Kong, Pokfulam, Hong Kong SAR, China

**Keywords:** COVID-19, family, fear, household income, psychological distress

## Abstract

**Background:**

The COVID-19 pandemic drives psychological distress. Previous studies have mostly focused on individual determinants but overlooked family factors. The present study aimed to examine the associations of individual and family factors with psychological distress, and the mediating effect of individual fear and the moderating role of household income on the above associations.

**Methods:**

We conducted a population-based cross-sectional survey on Chinese adults in Hong Kong from February to March 2021 (*N* = 2,251) to measure the independent variables of anti-epidemic fatigue, anti-epidemic confidence, individual and family members’ fear of COVID-19, and family well-being (range 0–10), and the dependent variable of psychological distress (through four-item Patient Health Questionnaire, range 0–4).

**Results:**

Hierarchical regression showed that anti-epidemic fatigue was positively (β = 0.23, 95% CI [0.18, 0.28]) while anti-epidemic confidence was negatively (β = −0.29, 95% CI [−0.36, −0.22]) associated with psychological distress. Family members’ fear of COVID-19 was positively (β = 0.11, 95% CI [0.05, 0.16]) while family well-being was negatively (β = −0.57, 95% CI [−0.63, −0.51]) associated with psychological distress. Structural equation model showed that individual fear mediated the above associations except for family well-being. Multi-group analyses showed a non-significant direct effect of anti-epidemic confidence and a slightly stronger direct effect of family well-being on psychological distress among participants with lower incomes, compared to those with higher incomes.

**Conclusion:**

We have first reported the double-edged effect of family context on psychological distress, with the positive association between family members’ fear of COVID-19 and psychological distress fully mediated by individual fear and the negative association between family well-being and psychological distress moderated by income level. Future studies are warranted to investigate how the contagion of fear develops in the family and how the inequality of family resources impacts family members’ mental health amid the pandemic.

## Introduction

Psychological distress has surged amid the COVID-19 pandemic (1, 2), and it is important to identify its risk and protective factors. Precautionary measures and vaccination may protect against COVID-19 (3), but no one is immune from the psychological distress it causes. Three meta-analyses showed the general population reported higher prevalence of psychological distress, particularly anxiety and depressive symptoms, during the COVID-19 pandemic compared to before the pandemic (4–6). Our cross-sectional study “Family amid COVID-19 survey 1” (FamCov-1) in May 2020 showed that Hong Kong adults who perceived harms rather than benefits from the pandemic reported low levels of happiness (7). However, previous studies have mainly focused on factors at the individual level, overlooking the importance of family, which serves as the immediate setting for individuals to shape and develop their cognitions and emotions (8, 9), particularly when family members are confined at home during the pandemic (10). Moreover, it is unclear about the potential mediator and moderator underlying the associations between individual and family factors and psychological distress.

The individual factors of anti-epidemic fatigue and anti-epidemic confidence contribute to psychological distress. The COVID-19 pandemic has continued for over 2 years, requiring continuous and varying efforts to control and cope with it, and people may feel exhausted and develop anti-epidemic fatigue (11) or gain positive beliefs and build up anti-epidemic confidence (12). The conservation of resources theory suggests that anti-epidemic fatigue may cause psychological distress with depleted mental resources to cope with stressors of the pandemic, while anti-epidemic confidence may improve mental health with increasing cognitive resources, including enriched anti-epidemic experiences and enhanced knowledge (13). As Hong Kong follows the “dynamic-zero” policy to minimize COVID-19 cases as much as possible (14), the general population are highly subject to the pandemic fatigue (15). Emerging evidence in Hong Kong has shown consequences of anti-epidemic fatigue, including lower adherence to control measures and depressive and anxiety symptoms (16, 17). However, like two sides of the same coin, anti-epidemic fatigue impedes the mitigation of the pandemic (18) while anti-epidemic confidence promotes the compliance with control measures (19). Compared with increasing studies on anti-epidemic fatigue, only one cross-sectional study showed a positive association between anti-epidemic confidence and mental health in Taiwan adults (12), leaving the scarcity of studies examining the psychological impacts of anti-epidemic confidence. Moreover, the extent to which anti-epidemic fatigue and anti-epidemic confidence are associated with psychological distress relative to family factors is unclear, as are the potential mediators underlying these associations.

Like the individual factors, the family may have a double-edged sword effect on psychological distress, with the transmission of fear through the family heating up symptoms of distress, and family well-being cooling them down. Suggested by Bronfenbrenner’s Ecological Systems Theory, family may influence an individual’s outcomes as the proximal context (20). On the one hand, dense living conditions and close family ties make Hong Kong families vulnerable to virus transmission, posing a much higher infection risk in family settings than in social and work settings (21). Based on the theory of emotion contagion, a high rate of household infection in Hong Kong may cause fear to prevail within families *via* their daily interactions (22). Although studies on SARS, H1N1, and COVID-19 support interdependent correlations of fear between parents and children and within couples (23–26), there is a lack of evidence on the mental health outcomes of family members’ fear of COVID-19. Only one study in Israel showed a non-significant association between family members’ fear of COVID-19 and psychological symptoms of stress, depression, and anxiety (27). Therefore, more evidence is needed to understand how family members’ fear of COVID-19 affects psychological distress. On the other hand, the family is often considered a “safe haven” that provides comfort and support to family members (28), with family well-being buffering the social disruption caused by the pandemic and facilitating mental adjustment (29). However, while the beneficial role of family well-being has been investigated in vulnerable family such as people with prior mental health disorders (30), the effect of family well-being in the general population is underexplored. Furthermore, no report has examined the relationship between family well-being and psychological distress during the COVID-19 pandemic.

Furthermore, fear may serve as a cognitive mechanism to mediate the associations between individual and family factors and psychological distress, which may be moderated by socio-demographic characteristics. Based on the transactional theory of stress and coping, fear may serve as an essential link in transmitting stress perception into psychological outcomes as a primary appraisal assessing the level of threat (31, 32). Individuals with an intense fear of COVID-19 have reported higher psychological distress (33), misperceived physical symptoms as signs of COVID-19 infection, and practiced excessive precautionary behaviors (34, 35). Our previous papers on FamCov-1 survey have also shown that those with higher fear of COVID-19 delayed doctor consultations and reported increased family conflict (36, 37). While accumulating studies showing the detrimental impacts of fear, it is unknown regarding the potentially mediating role of individual fear as a cognitive process. Besides, females, older people, those with less education and lower income may be more vulnerable during the pandemic (4, 38–40), which may cause difference in the underlying mediating mechanism. But it is no clear how such mediation mechanism *via* individual fear may differ by socio-demographic strata.

The present study aimed to (1) examine the associations of individual and family factors with psychological distress; (2) test the mediating effect of individual fear of COVID-19 on the above associations; and (3) investigate whether the mediating results differed by socio-demographic characteristics.

Based on the above theoretical framework and previous studies, we proposed the following hypotheses:

*Hypothesis 1a*: Anti-epidemic fatigue is positively associated with psychological distress.

*Hypothesis 1b*: Anti-epidemic confidence is negatively associated with psychological distress.

*Hypothesis 1c*: After controlling for individual factors, family members’ fear of COVID-19 is positively associated with psychological distress.

*Hypothesis 1d*: After controlling for individual factors, family well-being is negatively associated with psychological distress.

*Hypothesis 2*: Individual fear of COVID-19 mediates the above associations.

*Hypothesis 3*: The mediating results vary by socio-demographic characteristics.

## Methods

### Study design and participants

Under the Hong Kong Jockey Club Smart Family-Link Project, the current study was based on the population-based cross-sectional “Family amid COVID-19 survey 2” (FamCov-2) which was conducted using telephone interviews and online questionnaires from 22 February to 23 March 2021. We conducted the FamCov-1 in May 2020 when the second wave of COVID-19 outbreak was under control to assess individual attitudes, concerns, behaviors, and personal and family well-being, including fear (37), COVID-related information sharing (41), and delay in doctor consultation (36). Based on the FamCov-1, we conducted the second phase survey FamCov-2 in March 2021 when the fourth wave of COVID-19 outbreak was under control to assess the longer-term responses to the pandemic, including anti-epidemic fatigue and confidence (16), and adversity coping (42). The inclusion criteria of participants were those who (1) were Hong Kong residents aged 18 years or above, (2) could read or communicate using Cantonese, and (3) had landline or mobile telephone numbers or email accounts to contact. The Hong Kong Public Opinion Research Institute (HKPORI), a well-known local survey agency, was commissioned to conduct the survey.

The survey design and methods have been published (16, 42, 43). Specifically, this survey used a combination of probability and non-probability sampling methods. For the probability sampling, we adopted random telephone interview and probability-based online questionnaire survey. In both the landline and mobile telephone interviews, all telephone interviews were conducted by trained interviewers using random numbers generated by known prefixes assigned to telecommunication service providers. Invalid and working telephone numbers were identified by computers, and the remaining numbers were mixed in random to produce the number pool. A second-level sampling was only adopted in the landline telephone survey using the “next birthday rule” to select one eligible respondent among all those present in a household (i.e., select the qualified family member whose next birthday is nearest to the interview date). The online questionnaire survey invited HKPORI’s probability-panel, representative of the Hong Kong population, who were randomly selected in previous telephone surveys before this study. Totally 1,522 participants (62.9% out of the 2,420 valid telephone numbers) completed the telephone interview and 641 participants (14.9% out of the 4,311 probability-panel) completed the online survey, respectively. For the non-probability sampling, we adopted online questionnaire survey by inviting a convenience sample of HKPORI’s non-probability-panel (voluntarily registered members). Totally 5,372 participants out of the 44,514 non-probability-panel who opened the email invitations completed the online questionnaires.

The questionnaire comprises three subsets of questions on family communication, COVID-19 information, and COVID-19 influence. Each subset included core questions (which were present in all subsets) and subset-specific questions, and was randomly distributed to a third of all participants. The present analysis is based on the subset of COVID-19 influence, which was answered by 2,251 participants. Ethics approval was granted by the Institutional Review Board of the University of Hong Kong/Hospital Authority Hong Kong West Cluster (Reference number: UW 20-651). Oral consent for the telephone survey or written informed consent for the online survey was obtained from each participant before the survey commenced. To ensure confidentiality, we used anonymous questionnaire, stored data using a password, and restricted the use of data.

### Measurements

As assessment tools were not available to measure anti-epidemic fatigue, anti-epidemic confidence, and individual and family members’ fear of COVID-19 during our survey time, our research team designed a single item to efficiently and effectively assess each of these variables for Hong Kong community adults. Anti-epidemic fatigue was measured using a single question, “Do you think you have anti-epidemic fatigue?.” Anti-epidemic confidence was measured using a single question, “How much confidence do you have to cope with the COVID-19 pandemic?.” Individual fear and family members’ fear of COVID-19 were measured using a single question, “Has COVID-19 caused you/your family member(s) fear?,” as used in our previous studies (36, 37). Participants responded to each question using an 11-point scale ranging from 0 to 10, with higher scores indicating higher levels of the assessed variable.

Family well-being was measured using family health, harmony, and happiness (3Hs): three separate questions asked, “How healthy/harmonious/happy do you think your family is?” and were answered using an 11-point scale ranging from 0 to 10, which was developed and validated in our previous studies (44, 45). In addition to the three subscales of family 3Hs, we calculated a composite score of family well-being by dividing the sum of family 3Hs by three as in our previous studies, with higher scores indicating higher levels of family well-being (41, 42, 46). In the present study, the Cronbach’s *α* coefficient of the family 3Hs was 0.94.

Psychological distress was measured using the four-item Patient Health Questionnaire (PHQ-4), including 2 two-item subscales measuring anxiety and depressive symptoms (47). The Chinese version of PHQ-4 has shown good reliability and validity (48, 49). Participants indicated the frequency of the described symptoms over the previous 2 weeks using a 4-point Likert scale from 0 (not at all) to 3 (nearly every day). The total scores for PHQ-4 ranged from 0 to 12, with higher total scores indicating higher psychological distress. In the present study, Cronbach’s *α* coefficient was 0.88 for the PHQ-4, and 0.82 and 0.79 for the subscales measuring anxiety and depressive symptoms, respectively.

We collected information on socio-demographic characteristics, including sex, age, education level, monthly household income, and number of cohabitants. We recoded age as “18–44 years” or “≥ 45 years” and education level as “secondary or below” or “tertiary.” Monthly household income per person (income divided by household size) was calculated and dichotomized as “lower” or “higher” with reference to the size-specific median monthly household income in Hong Kong’s census statistics (50). Except for the socio-demographic characteristics, each variable was treated as continuous variable without cut-off value.

### Statistical analyses

Descriptive statistics of socio-demographic characteristics were computed and weighted by sex, age group, and education level based on data from the 2019 Hong Kong census (51). Hierarchical regression analysis was used to examine the associations of individual and family factors with psychological distress (Hypotheses 1a to 1d), using a regression coefficient (beta, β) with 95% confidence interval (CIs) to estimate the strength and direction of the associations. To be specific, in Step 1, socio-demographic characteristics were added to examine their associations with psychological distress. In Step 2, individual factors (i.e., anti-epidemic fatigue and confidence) were added to examine the associations between individual factors and psychological distress beyond the effects of socio-demographic characteristics. In Step 3, family factors (i.e., family members’ fear of COVID-19 and family well-being) were added to examine the associations between family factors and psychological distress beyond the effects of individual factors and socio-demographic characteristics. Finally, in Step 4, individual fear was added to preliminarily examine its mediating effect on the associations aforementioned (52).

Structural equation model (SEM) was used to examine the mediating effect (i.e., indirect effect) of individual fear of COVID-19 by decomposing the aforementioned associations into direct and indirect effects (Hypothesis 2) (53). Missing values were handled using full information maximum likelihood estimation. Standardized coefficients and bias-corrected (BC) 95% CI of the direct and indirect effects were estimated by the maximum likelihood and bootstrap methods with 1,000 replications, respectively. We examined the fit indices of the SEM, including (1) root mean square error of approximation (RMSEA) and standardized root mean square residual (SRMR), with RMSEA < 0.06 and SRMR < 0.08 considered as good fit indices; and (2) comparative Fit Index (CFI) and Tucker-Lewis index (TLI), with CFI > 0.95 and TLI > 0.95 considered as good fit indices (54, 55).

Multi-group analysis was used to investigate whether the SEM results differed according to socio-demographic characteristics (Hypothesis 3). To be specific, we conducted four multi-group analyses to test whether the SEM results were equal across sex (male vs. female), age group (18–44 years vs. ≥ 45 years), education level (secondary or below vs. tertiary), and monthly household income per person (lower vs. higher), including three steps for each multi-group analysis (56, 57). First, an unconstrained model (all path coefficients were freely estimated across different groups) and a fully constrained model (all path coefficients were constrained to equality across different groups) were compared using likelihood-ratio tests. Second, if the result of the model comparison indicated a statistically significant difference, we released the constraint of the specific path coefficient with the largest modification index in the fully constrained model and obtained a partially constrained model. Third, we compared the partially constrained model with the unconstrained model. The last two steps were repeated until there was no statistically significant difference between the partially constrained and unconstrained models.

In hierarchical regression analysis, we used the total scores of PHQ-4 and the composite score of family 3Hs to indicate psychological distress and family well-being, respectively. In SEM and multi-group analysis, we used the two PHQ-4 subscales measuring anxiety and depressive symptoms to indicate the latent variable of psychological distress, and the three family 3Hs subscales to indicate the latent variable of family well-being. We used STATA/SE 17.0 and the “lavaan” package in R 4.2.1 for these statistical analyses (58). Two-sided *p* < 0.05 was considered statistically significant.

## Results

### Socio-demographic characteristics of the participants

Table 1 shows that, after weighting, about half of the participants were female (52.6%). Most participants were 45 years old or above (58.7%), had secondary education level or below (61.2%) and lower monthly household income per person than the median level in Hong Kong’s 2019 census statistics (56.0%). The mean number of cohabitants was 2.20 ± 1.35. For socio-demographic characteristics, the differences between unweighted and weighted data in education level reached a medium level of effect size (Cramer’s V = 0.37), and in monthly household income per person reached a small level of effect size (Cramer’s V = 0.13). However, for individual and family factors, the weighted data showed little difference with the unweighted data.

**Table 1:** Descriptive statistics of socio-demographic characteristics, individual and family factors.

	Unweighted	Weighted^a^	Effect size^b^
**Sex, *n* (%, 95 CI)**			
Male	1,078 (47.9, 45.8–50.0)	1,059 (47.4, 43.9–50.8)	0.01
Female	1,173 (52.1, 50.0–54.2)	1,177 (52.6, 49.2–56.1)	
**Age group (years), *n* (%, 95 CI)**			
18–44	1,105 (49.1, 47.1–51.2)	923 (41.3, 38.0–44.7)	0.08
≥45	1,144 (50.9, 48.8–52.9)	1,311 (58.7, 55.3–62.0)	
**Education level, *n* (%, 95 CI)**			
Secondary or below	552 (24.7, 22.9–26.5)	1,363 (61.2, 58.1–64.3)	0.37
Tertiary	1,686 (75.3, 73.5–77.1)	863 (38.8, 35.7–41.9)	
**Monthly household income per person, *n* (%, 95 CI)**			
Lower (<median)	834 (42.9, 40.7–45.1)	1,105 (56.0, 52.4–60.0)	0.13
Higher (≥median)	1,111 (57.1, 54.9–59.3)	867 (44.0, 40.4–47.6)	
Number of cohabitants, Mean (SD)	2.20 (1.38)	2.20 (1.35)	0.001
Anti-epidemic fatigue^c^, Mean (SD)	5.94 (2.61)	5.75 (2.62)	0.07
Anti-epidemic confidence^c^, Mean (SD)	6.83 (1.77)	6.76 (1.85)	0.04
Individual fear of COVID-19^c^, Mean (SD)	5.00 (2.46)	5.01 (2.43)	0.001
Family members’ fear of COVID-19^c^, Mean (SD)	5.48 (2.22)	5.43 (2.16)	0.02
Family well-being^d^, Mean (SD)	6.45 (1.93)	6.47 (1.89)	0.01

a Weighted by sex, age group, and education level based on data from the 2019 Hong Kong census.

b Effect size: Cramer’s V was used to calculate the effect size of differences between pairs of proportions for the categorical variable: 0.10–0.30, small; 0.30–0.50, medium; ≥0.50, large. Cohen’s *d* for the continuous variable: 0.20–0.50, small; 0.50–0.80, medium; ≥0.80, large.

c A single item ranges from 0 to 10, with higher scores indicating higher levels.

d Family well-being is indicated by the composite score calculated by dividing the sum of family health, family harmony, and family happiness by three, ranging from 0 to 10 with higher scores indicating higher levels of family well-being.

Participants with missing values were excluded.

### Associations of individual and family factors with psychological distress

Supplementary Table 1 shows that all variables of individual and family factors and psychological distress had significant bivariate correlations, except for the association (1) between individual fear of COVID-19 and family well-being, and (2) between family member’s fear of COVID-19 and family well-being.

Table 2 shows the results of hierarchical regression analyses. When adding socio-demographic characteristics in Step 1, age of 45 years or above (β = −1.34, 95% CI [−1.60, −1.07], *p* < 0.001), tertiary education (β = 0.37, 95% CI [0.04, 0.69], *p* = 0.03), and higher monthly household income per person (β = −0.37, 95% CI [−0.64, −0.10], *p* = 0.007) were associated with higher psychological distress. Socio-demographic characteristics explained 7% of the variance of psychological distress. When adding individual factors in Step 2, anti-epidemic fatigue was positively associated with psychological distress (β = 0.23, 95% CI [0.18, 0.28], *p* < 0.001), while anti-epidemic confidence was negatively associated with psychological distress (β = −0.29, 95% CI [−0.36, −0.22], *p* < 0.001), supporting H1a and H1b, respectively.

**Table 2:** Results of hierarchical regression examining the associations between individual and family factors with psychological distress.

	Psychological distress^a^			
	β (95% CI)			
	Step 1	Step 2	Step 3	Step 4
**Socio-demographic characteristics**				
Sex				
Male	1	1	1	1
Female	0.07 (−0.18, 0.32)	−0.07 (−0.30, 0.17)	0.04 (−0.18, 0.27)	−0.01 (−0.23, 0.22)
Age group (years)				
18–44	1	1	1	1
≥45	−1.34 (−1.60, −1.07)^***^	−1.20 (−1.45, −0.94)^***^	−0.80 (−1.04, −0.56)^***^	−0.80 (−1.04, −0.56)^***^
Education level				
Secondary or below	1	1	1	1
Tertiary	0.37 (0.04, 0.69)^*^	0.24 (−0.08, 0.55)	0.07 (−0.22, 0.36)	0.07 (−0.22, 0.36)
Monthly household income per person				
Lower (<median)	1	1	1	1
Higher (≥median)	−0.37 (−0.64, −0.10)^**^	−0.29 (−0.55, −0.03)^*^	−0.09 (−0.33, 0.15)	−0.09 (−0.33, 0.15)
Number of cohabitants	−0.07 (−0.16, 0.03)	−0.08 (−0.17, 0.02)	−0.05 (−0.13, 0.03)	−0.05 (−0.13, 0.04)
**Individual factors**				
Anti-epidemic fatigue^b^		0.23 (0.18, 0.28)^***^	0.18 (0.14, 0.23)^***^	0.17 (0.13, 0.22)^***^
Anti-epidemic confidence^b^		−0.29 (−0.36, −0.22)^***^	−0.12 (−0.18, −0.05)^***^	−0.10 (−0.16, −0.03)^**^
**Family factors**				
Family members’ fear of COVID-19^b^			0.11 (0.05, 0.16)^***^	0.05 (−0.02, 0.11)
Family well-being^c^			−0.57 (−0.63, −0.51)^***^	−0.58 (−0.64, −0.51)^***^
**Mediating factor**				
Individual fear of COVID-19^b^				0.10 (0.03, 0.16)^**^
R-square	0.07	0.15	0.28	0.29
R-square change		0.08^***^	0.13^***^	0.01^**^

a Psychological distress is indicated by the total score of four-item Patient Health Questionnaire, ranging from 0 to 12 with higher scores indicating higher psychological distress.

b A single item ranges from 0 to 10, with higher scores indicating higher levels.

c Family well-being is indicated by the composite score calculated by dividing the sum of family health, family harmony, and family happiness by three, ranging from 0 to 10 with higher scores indicating higher levels of family well-being.

^*^p < 0.05, ^**^p < 0.01, ^***^p < 0.001.

When adding family factors in Step 3, family members’ fear of COVID-19 was positively associated with psychological distress (β = 0.11, 95% CI [0.05, 0.16], *p* < 0.001), while family well-being was negatively associated with psychological distress (β = −0.57, 95% CI [−0.63, −0.51], *p* < 0.001), supporting H1c and H1d, respectively. Individual factors accounted for 8% of the variance of psychological distress, and family factors accounted for an additional 13%. Using the subscales of PHQ-4 measuring anxiety and depressive symptoms as two separate outcomes in regression analyses, we found that individual and family factors remained significant, although with smaller coefficients compared to those found when using the total scores of PHQ-4 scores (see Supplementary Tables 2, 3).

### Mediating effects of individual fear of COVID-19

When individual fear of COVID-19 was added in Step 4, the coefficient and *p*-value of anti-epidemic confidence both appeared to decrease slightly, suggesting that individual fear of COVID-19 might have a partial mediating effect; the association between family members’ fear of COVID-19 and psychological distress became non-significant, suggesting that individual fear of COVID-19 might have a full mediating effect.

The hypothesized model with the mediating effect of individual fear of COVID-19 showed good fit indices: χ^2^ = 157.51, *df* = 29, RMSEA = 0.04, SRMR = 0.01, CFI = 0.99, TLI = 0.98. Supplementary Figure 1 shows the path diagram with the standardized regression coefficients of the hypothesized model. We adjusted sex, age group, education level, monthly household income per person, and number of cohabitants in this model.

Table 3 shows that, for individual factors, the mediating effect of individual fear of COVID-19 was statistically significant on the association between anti-epidemic fatigue and psychological distress (standardized β = 0.010, BC 95% CI [0.003, 0.020], *p* = 0.011), and the association between anti-epidemic confidence and psychological distress (standardized β = −0.013, BC 95% CI [−0.026, −0.005], *p* = 0.009), respectively. Given both direct effects were statistically significant, the mediating effect was partial (proportion mediated: 5.92% and 18.31%, respectively). For family factors, the mediating effect of individual fear of COVID-19 was statistically significant on the association between family members’ fear of COVID-19 and psychological distress (standardized β = 0.046, BC 95% CI [0.016, 0.082], *p* = 0.005), but not statistically significant on the association between family well-being and psychological distress (standardized β = 0.004, BC 95% CI [−0.001, 0.010], *p* = 0.089), partly supporting our H2. Given the direct effect of family members’ fear of COVID-19 on psychological distress was not statistically significant, the mediating effect was full.

**Table 3:** Direct and indirect effects in the structural equation model.

	Standardized coefficients (β)	BC^a^ 95% CI	*p*-values
**Direct effects**			
Anti-epidemic fatigue^b^ → psychological distress^c^	0.159	(0.116, 0.201)	< 0.001
Anti-epidemic confidence^b^ → psychological distress^c^	−0.058	(−0.103, −0.009)	0.017
Family members’ fear of COVID-19^b^ → Psychological distress^c^	0.021	(−0.033, 0.075)	0.444
Family well-being^d^ → psychological distress^c^	−0.460	(−0.505, −0.414)	< 0.001
**Indirect effects (*via* individual fear of COVID-19)**			
Anti-epidemic fatigue^b^ → individual fear of COVID-19^b^ → psychological distress^c^	0.010	(0.003, 0.020)	0.011
Anti-epidemic confidence^b^ → individual fear of COVID-19^b^ → psychological distress^c^	−0.013	(−0.026, −0.005)	0.009
Family members’ fear of COVID-19^b^ → individual fear of COVID-19^b^ → psychological distress^c^	0.046	(0.016, 0.082)	0.005
Family well-being^d^ → individual fear of COVID-19^b^ → psychological distress^c^	0.004	(−0.001, 0.010)	0.089

a BC: Bias-corrected (BC 95% CI corrects for the bias and skewness in the distribution of bootstrap method).

b A single item ranges from 0 to 10, with higher scores indicating higher levels.

c Psychological distress is a latent variable indicated by anxiety and depressive symptoms (each ranging from 0 to 6, with higher scores indicating higher levels).

d Family well-being is a latent variable indicated by family health, family harmony, and family happiness (each ranging from 0 to 10, with higher scores indicating higher levels).

This model was adjusted by sex, age group, education level, monthly household income per person, and number of cohabitants.

### Multi-group analyses to examine socio-demographic differences

As Supplementary Table 4 shows, the comparisons of multiple-group models stratified by sex [Δχ^2^_(17)_ = 23.21, *p* = 0.142], age group [Δχ^2^_(17)_ = 24.45, *p* = 0.108], and education level [Δχ^2^_(17)_ = 17.73, *p* = 0.406] indicated that the SEM results did not differ by these characteristics. However, when stratified by monthly household income per person, the model comparison showed that the fully constrained model fit worse than the unconstrained model, indicating significant differences in one or more path coefficients across the groups [Δχ^2^_(17)_ = 29.72, *p* = 0.028]. Supplementary Table 5 shows that participants in the lower income group reported lower anti-epidemic fatigue, anti-epidemic confidence, and family well-being than those in the higher income group.

We repeated the iterative process to obtain a final partially constrained model (χ^2^ = 464.04, *df* = 88, RMSEA = 0.07, SRMR = 0.07, CFI = 0.96, TLI = 0.95), which was not statistically significantly different from the unconstrained model [Δχ^2^_(15)_ = 24.39, *p* = 0.059]. In the final model, we released the constraints on two path coefficients in the fully constrained model: (1) the direct effect of anti-epidemic confidence on psychological distress, and (2) the direct effect of family well-being on psychological distress, indicating that these two path coefficients were significantly different across lower and higher income groups. Supplementary Figure 2 shows that the direct effect of anti-epidemic confidence on psychological distress was not statistically significant in the lower income group (standardized β = −0.03, *p* = 0.359) but was statistically significant in the higher income group (standardized β = −0.10, *p* = 0.001). Besides, the direct effect of family well-being on psychological distress was slightly stronger in the lower income group (standardized β = −0.49, *p* < 0.001) than in the higher income group (standardized β = −0.42, *p* < 0.001).

## Discussion

We have first shown, by extending beyond the individual factors and finding that anti-epidemic fatigue showed a positive association with psychological distress while anti-epidemic confidence showed a negative association, that the family context appeared to be a double-edged sword, with psychological distress positively associated with family members’ fear and negatively associated with family well-being. We have further shown that individual fear of COVID-19 mediated all the above associations except for family well-being. Disparities by different monthly household income per person were evident, with a non-significant direct effect of anti-epidemic confidence and a slightly stronger direct effect of family well-being on psychological distress among those with lower incomes, compared to those with higher incomes. Our study provides some new insight into how the family context can lead to psychological distress, with one for family members’ fear of COVID-19 fully mediated by individual fear and the other for family well-being moderated by income level.

As expected, anti-epidemic fatigue was positively associated with psychological distress, with a mediating effect of individual fear of COVID-19. These two findings can be explained by the process of self-regulation (59), through which individuals take actions to cope with changing situations to achieve their goals (e.g., pandemic control), and evaluate whether their efforts have achieved their goals through external feedback. If people try to follow the government recommended measures to cope with the pandemic, but continuously receive negative feedback about fluctuating and rising infection cases and deaths, they may feel a sense of failure and helplessness, followed by psychological distress. The negative feedback of the unsolved pandemic may also evoke the emotion of fear, as individuals may feel that their abilities are limited and the measures they have adopted are ineffective in returning to normal life despite huge efforts and sacrifices.

Our study found that anti-epidemic confidence was negatively associated with psychological distress, mediated by individual fear of COVID-19, particularly among those with higher incomes. These findings can be explained by the extended parallel process model, which proposes that fear is determined by levels of perceived self-efficacy and response efficacy (60). Those who have more confidence in handling the pandemic have higher perceived efficacy, with greater belief in their abilities and the effectiveness of preventive behaviors; they thus feel less fear and lower psychological distress. As the prolonged pandemic has led to adverse impacts on the economy, including reduced salaries and increased unemployment (61), those with lower incomes not only reported lower anti-epidemic confidence, but also showed a non-significant direct effect of anti-epidemic confidence on psychological distress, compared with those with higher incomes. This is understandable, as those with financial hardship have great difficulties in acquiring adequate supplies for daily life, personal protective materials, and medical services to build up their confidence when handling the pandemic.

Our study showed that family members’ fear of COVID-19 was positively associated with psychological distress *via* the full mediating effect of individual fear of COVID-19. Facing with uncertain situation at early wave of the outbreak, our FamCov-1 study showed that the general population reported a moderate level of fear and found that individuals with higher levels of fear reported lower happiness (37). Extending the FamCov-1’s findings, the current study showed that perceived fear of family members strengthened one’s own fear, and then worsened one’s mental health problems. Future studies using longitudinal designs are warranted to investigate how fear contagion develops within families and affects family members, especially among healthcare workers and public service providers with higher exposure to the virus. Interestingly, compared to participants in the higher income group, although those in the lower income group reported lower levels of family well-being, the direct negative effect of family well-being on psychological distress was slightly stronger among them. Based on studies showing the importance of family dynamics in managing COVID-19 stressors (62, 63), our findings support and extend previous findings by showing that family well-being nurtured in family dynamics could protect individuals from psychological distress. In addition to family well-being, further studies on the inequity of other family resources between people with lower and higher economic status are needed to understand the effects on mental health, as our findings suggest that a healthy, harmonious, and happy family may have a slightly stronger buffering effect on psychological distress among those with lower incomes. Besides, we also found that the mediating effect of individual fear of COVID-19 on the association between family well-being and psychological distress was non-significant. This may be explained by other potential mediators not identified and included in previous studies and our present study, such as uncertainty and coping strategies (64, 65).

Our study has some limitations. Firstly, the cross-sectional study design limits causal inference, as the temporal sequence of variables were unclear, and the potential confounding variables were unknown and not being controlled. Secondly, family factors, including family members’ fear and family well-being, were perceived and self-reported, and may be subject to subjective bias. Thirdly, as valid and brief tools to assess anti-epidemic fatigue, anti-epidemic confidence, and family members’ fear of COVID-19 were not available when we conducted the survey, the psychometric properties of our single-item measures need to be further investigated. Moreover, as our sample included more participants with higher education and household incomes, their understanding of our measurements may differ from the general population, which may cause information bias. However, giving responses to simple and direct questions may have allowed participants to reveal their genuine feelings and thoughts quickly. Finally, this study might have selection bias because only telephone interviews and online questionnaires (*via* email invitations) were used for data collection. Therefore, our findings may have limited generalizability for not covering the significant volume of non-respondents (e.g., the older might have less access to or less preference for being contacted through telephone or email). Nevertheless, there was little difference in our study variables when we compared unweighted and weighted results by sex, age group, and education level based on data from the 2019 Hong Kong census (see Table 1).

## Conclusion

Beyond investigating individual factors of anti-epidemic fatigue and anti-epidemic confidence, this study is the first to report the double-edged effect of family context on psychological distress. Specifically, the positive association between family members’ fear and psychological distress was fully mediated by individual fear of COVID-19, and the negative association between family well-being and psychological distress was strengthened among those with lower incomes than those with higher incomes. Future studies are warranted to investigate how fear contagion develops in the family and how the inequality of family resources impacts family members’ mental health amid the pandemic.

## Data availability statement

The datasets presented in this article are not readily available because the sharing of data to third parties was not mentioned in the subjects’ consent. The dataset supporting the current study is available on request from the corresponding authors. Requests to access the datasets should be directed to NY, nancy.yu@cityu.edu.hk; MW, mpwang@hku.hk.

## Ethics statement

The studies involving human participants were reviewed and approved by the Institutional Review Board of the University of Hong Kong/Hospital Authority Hong Kong West Cluster (Reference number: UW 20–651). The patients/participants provided their written informed consent to participate in this study.

## Author contributions

BC, WG, NY, and TL: conceptualization and methodology. BC: formal analysis and writing—original draft. WG: data curation. BC, WG, AL, SS, SH, NY, MW, and TL: writing—review and editing. SH, MW, and TL: resources, supervision, and funding acquisition. All authors contributed to the article and approved the submitted version.

## Funding

This research was funded by the Hong Kong Jockey Club Charities Trust.

## Conflict of interest

The authors declare that the research was conducted in the absence of any commercial or financial relationships that could be construed as a potential conflict of interest.

## Publisher’s note

All claims expressed in this article are solely those of the authors and do not necessarily represent those of their affiliated organizations, or those of the publisher, the editors and the reviewers. Any product that may be evaluated in this article, or claim that may be made by its manufacturer, is not guaranteed or endorsed by the publisher.
