# Supplementary material for: Family context as a double-edged sword for psychological distress amid the COVID-19 pandemic with the mediating effect of individual fear and the moderating effect of household income
Source: Front Public Health. 2023 Mar 23;11:1109446. doi: 10.3389/fpubh.2023.1109446 (PMC10076528; doi:10.3389/fpubh.2023.1109446)
Supplement: Supplementary file 1 [file Table_1.docx]

Supplementary Material

Family context as a double-edged sword for psychological distress amid the COVID-19 pandemic with the mediating effect of individual fear and the moderating effect of household income

Bowen Chen^1^, Weijie Gong^2,3^, Agnes Yuen Kwan Lai^4^, Shirley Man Man Sit^3, 4^, Sai Yin Ho^3^, Nancy Xiaonan Yu^1*^, Man Ping Wang^4*^, Tai Hing Lam^3^

^1^Department of Social and Behavioural Sciences, City University of Hong Kong, Hong Kong SAR, China

^2^ Department of General Practice, Medical School, Shenzhen University, Shenzhen, China

^3^ School of Public Health, The University of Hong Kong, Hong Kong SAR, China

^4^ School of Nursing, The University of Hong Kong, Hong Kong SAR, China

*** Correspondence:** Nancy Xiaonan Yu: [nancy.yu@cityu.edu.hk](mailto:nancy.yu@cityu.edu.hk); Man Ping Wang: [mpwang@hku.hk](mailto:mpwang@hku.hk).

**Supplementary Table1.** Descriptive statistics and bivariate correlations of individual and family factors, and psychological distress

| **Variables** | **Mean ± SD** | **1** | **2** | **3** | **4** | **5** | **6** | **7** | **8** |
| --- | --- | --- | --- | --- | --- | --- | --- | --- | --- |
| 1. Anti-epidemic fatigue^a^ | 5.94 ± 2.61 | 1.00 |  |  |  |  |  |  |  |
| 2. Anti-epidemic confidence^a^ | 6.83 ± 1.77 | -0.11*** | 1.00 |  |  |  |  |  |  |
| 3. Individual fear of COVID-19^a^ | 5.00 ± 2.46 | 0.32*** | -0.26*** | 1.00 |  |  |  |  |  |
| 4. Family members’ fear of COVID-19^a^ | 5.48 ± 2.22 | 0.30*** | -0.14*** | 0.65*** | 1.00 |  |  |  |  |
| Family well-being |  |  |  |  |  |  |  |  |  |
| 5. Family health^a^ | 6.36 ± 2.02 | -0.11*** | 0.25*** | -0.01 | -0.01 | 1.00 |  |  |  |
| 6. Family harmony^a^ | 6.62 ± 2.07 | -0.07*** | 0.22*** | 0.01 | 0.01 | 0.81*** | 1.00 |  |  |
| 7. Family happiness^a^ | 6.36 ± 2.03 | -0.10*** | 0.23*** | -0.01 | 0.01 | 0.85*** | 0.86*** | 1.00 |  |
| Psychological distress |  |  |  |  |  |  |  |  |  |
| 8. Anxiety symptoms^b^ | 3.72 ± 1.52 | 0.23*** | -0.19*** | 0.18*** | 0.15*** | -0.41*** | -0.35*** | -0.44*** | 1.00 |
| 9. Depressive symptoms^b^ | 3.55 ± 1.53 | 0.23*** | -0.19*** | 0.11*** | 0.09*** | -0.43*** | -0.38*** | -0.47*** | 0.75*** |

^a^A single item ranges from 0 to 10, with higher scores indicating higher levels.

^b^The subscale of four-item Patient Health Questionnaire measuring anxiety/depressive symptoms, ranging from 0 to 6 with higher scores indicating higher anxiety/depressive symptoms.

****P* < 0.001.

**Supplementary Table 2.** Results of hierarchical regression examining the associations between individual and family factors with anxiety symptoms

|  | Anxiety symptoms^a^ | | | |
| --- | --- | --- | --- | --- |
|  | β (95% CI) | | | |
|  | Step 1 | Step 2 | Step 3 | Step 4 |
| **Socio-demographic characteristics** | |  |  |  |
| Sex |  |  |  |  |
| Male | 1 | 1 | 1 | 1 |
| Female | 0.10 (-0.04, 0.23) | 0.03 (-0.10, 0.16) | 0.09 (-0.03, 0.21) | 0.05 (-0.07, 0.18) |
| Age group (years) |  |  |  |  |
| 18–44 | 1 | 1 | 1 | 1 |
| ≥ 45 | -0.62 (-0.76, -0.47)*** | -0.55 (-0.69, -0.41)*** | -0.34 (-0.47, -0.21)*** | -0.34 (-0.47, -0.21)*** |
| Education level |  |  |  |  |
| Secondary or below | 1 | 1 | 1 | 1 |
| Tertiary | 0.24 (0.07, 0.42)** | 0.18 (0.01, 0.35)* | 0.09 (-0.07, 0.25) | 0.09 (-0.07, 0.25) |
| Monthly household income per person | |  |  |  |
| Lower (< Median) | 1 | 1 | 1 | 1 |
| Higher (≥ Median) | -0.15 (-0.29, -0.01)* | -0.11 (-0.26, 0.03) | -0.01 (-0.14, 0.12) | -0.01 (-0.14, 0.12) |
| Number of cohabitants | -0.02 (-0.08, 0.03) | -0.03 (-0.08, 0.02) | -0.02 (-0.07, 0.03) | -0.02 (-0.06, 0.03) |
| **Individual factors** |  |  |  |  |
| Anti-epidemic fatigue^b^ |  | 0.11 (0.08, 0.13)*** | 0.09 (0.06, 0.11)*** | 0.08 (0.05, 0.10)*** |
| Anti-epidemic confidence^b^ | | -0.29 (-0.36, -0.22)*** | -0.15 (-0.19,-0.11)*** | -0.07 (-0.10, -0.03)*** |
| **Family factors** |  |  |  |  |
| Family members’ fear of COVID-19^b^ | |  |  | 0.07 (0.05, 0.10)*** |
| Family well-being^c^ |  |  | -0.28 (-0.32,-0.25)*** | -0.29 (-0.32, -0.25)*** |
| **Mediating factor** |  |  |  |  |
| Individual fear of COVID-19^b^ | |  |  |  |
| R-square | 0.05 | 0.13 | 0.24 | 0.25 |
| R-square change |  | 0.08*** | 0.11*** | 0.01*** |

^a^ Anxiety symptoms are indicated by the subscale of four-item Patient Health Questionnaire measuring anxiety symptoms, ranging from 0 to 6 with higher scores indicating higher anxiety symptoms. ^b^ A single item ranges from 0 to 10, with higher scores indicating higher levels. ^c^ Family well-being is indicated by the composite score calculated by dividing the sum of family health, family harmony, and family happiness by three, ranging from 0 to 10 with higher scores indicating higher levels of family well-being.

**P* < 0.05, ***P* < 0.01, ****P* < 0.001.

**Supplementary Table 3.** Results of hierarchical regression examining the associations between individual and family factors with depressive symptoms

|  | Depressive symptoms^a^ | | | |
| --- | --- | --- | --- | --- |
|  | β (95% CI) | | | |
|  | Step 1 | Step 2 | Step 3 | Step 4 |
| **Socio-demographic characteristics** | |  |  |  |
| Sex |  |  |  |  |
| Male | 1 | 1 | 1 | 1 |
| Female | -0.03 (-0.16, 0.10) | -0.10 (-0.22, 0.03) | -0.04 (-0.16, 0.08) | -0.06 (-0.18, 0.06) |
| Age group (years) |  |  |  |  |
| 18–44 | 1 | 1 | 1 | 1 |
| ≥ 45 | -0.74 (-0.88, -0.60)*** | -0.67 (-0.81, -0.53)*** | -0.46 (-0.59, -0.33)*** | -0.46(-0.59, -0.33)*** |
| Education level |  |  |  |  |
| Secondary or below | 1 | 1 | 1 | 1 |
| Tertiary | 0.12 (-0.05, 0.29) | 0.05 (-0.12, 0.22) | -0.02 (-0.17, 0.14) | -0.02 (-0.17, 0.14) |
| Monthly household income per person | |  |  |  |
| Lower (< Median) | 1 | 1 | 1 | 1 |
| Higher (≥ Median) | -0.21 (-0.35, -0.06)** | -0.18 (-0.32, -0.04)* | -0.07 (-0.20, 0.06) | -0.07 (-0.20, 0.06) |
| Number of cohabitants | -0.04 (-0.09, 0.01) | -0.04 (-0.09, 0.01) | -0.03 (-0.08, 0.01) | -0.03 (-0.08, 0.02) |
| **Individual factors** |  |  |  |  |
| Anti-epidemic fatigue^b^ |  | 0.11 (0.09, 0.14)*** | 0.10 (0.07, 0.12)*** | 0.09 (0.07, 0.12)*** |
| Anti-epidemic confidence^b^ | | -0.29 (-0.36, -0.22)*** | -0.14 (-0.17, -0.10)*** | -0.05 (-0.09, -0.02)** |
| **Family factors** |  |  |  |  |
| Family members’ fear of COVID-19^b^ | |  |  | 0.03 (0.01, 0.06)** |
| Family well-being^c^ |  |  | -0.29 (-0.32,-0.25)*** | -0.29 (-0.32, -0.26)*** |
| **Mediating factor** |  |  |  |  |
| Individual fear of COVID-19^b^ | |  |  |  |
| R-square | 0.06 | 0.13 | 0.25 | 0.26 |
| R-square change |  | 0.07*** | 0.12*** | 0.01* |

^a^ Depressive symptoms are indicated by the subscale of four-item Patient Health Questionnaire measuring depressive symptoms, ranging from 0 to 6 with higher scores indicating higher anxiety symptoms. ^b^ A single item ranges from 0 to 10, with higher scores indicating higher levels. ^c^ Family well-being is indicated by the composite score calculated by dividing the sum of family health, family harmony, and family happiness by three, ranging from 0 to 10 with higher scores indicating higher levels of family well-being.

**P* < 0.05, ***P* < 0.01, ****P* < 0.001.

**Supplementary Table 4.** Model fit indices of multiple structural equation models stratified by sex, age group, education level, and monthly household income per person

|  | **χ^2^** | ***df*** | **RMSEA** | **SRMR** | **CFI** | **TLI** | **Model comparisons** |
| --- | --- | --- | --- | --- | --- | --- | --- |
| Criteria of goodness-of-fit | - | - | < 0.06 | < 0.08 | > 0.95 | > 0.95 |  |
| Multiple-group models |  |  |  |  |  |  |  |
| Stratified by sex |  |  |  |  |  |  | Δχ^2^_(17)_ = 23.21 (*P* = 0.142) |
| Unconstrained model | 431.10 (*P* < 0.001) | 73 | 0.07 | 0.07 | 0.96 | 0.94 |  |
| Fully constrained model | 454.31 (*P* < 0.001) | 90 | 0.07 | 0.07 | 0.96 | 0.95 |  |
| Stratified by age group |  |  |  |  |  |  | Δχ^2^_(17)_ = 24.45 (*P* = 0.108) |
| Unconstrained model | 416.02 (*P* < 0.001) | 73 | 0.07 | 0.07 | 0.96 | 0.94 |  |
| Fully constrained model | 440.47 (*P* < 0.001) | 90 | 0.06 | 0.07 | 0.96 | 0.95 |  |
| Stratified by education level |  |  |  |  |  |  | Δχ^2^_(17)_ = 17.73 (*P* = 0.406) |
| Unconstrained model | 445.76 (*P* < 0.001) | 73 | 0.07 | 0.07 | 0.96 | 0.94 |  |
| Fully constrained model | 463.49 (*P* < 0.001) | 90 | 0.07 | 0.07 | 0.96 | 0.95 |  |
| Stratified by monthly household income per person | |  |  |  |  |  | Δχ^2^_(17)_ = 29.72 (*P* = 0.028) |
| Unconstrained model | 439.65 (*P* < 0.001) | 73 | 0.07 | 0.07 | 0.96 | 0.94 |  |
| Fully constrained model | 469.37 (*P* < 0.001) | 90 | 0.07 | 0.07 | 0.96 | 0.95 |  |

Root mean square error of approximation (RMSEA) and standardized root mean square residual (SRMR): RMSEA and SRMR assess how far a hypothesized model is from a perfect model, with 0 indicating the best fit and higher values indicating the lack of fit. RMSEA < 0.06 and SRMR < 0.08 are considered as good fit indices. Comparative Fit Index (CFI) and Tucker-Lewis index (TLI): CFI and TLI indicate how well a hypothesized model fits the data relative to a null model (a null model assumes that sampling error alone explains the covariation among the observed measures). CFI > 0.95 and TLI > 0.95 are considered as good fit indices. Model comparisons are based on the results of likelihood-ratio tests.

**Supplementary Table 5.** Descriptive statistics and comparisons of individual and family factors, and psychological distress in lower and higher monthly household income groups

|  | Mean ± SD | | *P*-values | Effect size^b^ |
| --- | --- | --- | --- | --- |
|  | Monthly household income per person^a^ | |  |  |
|  | Lower | Higher |  |  |
| Anti-epidemic fatigue^c^ | 5.79 ± 2.74 | 6.12 ± 2.50 | 0.007 | 0.12 |
| Anti-epidemic confidence^c^ | 6.61 ± 1.89 | 7.05 ± 1.65 | <0.001 | 0.25 |
| Individual fear of COVID-19^c^ | 5.00 ± 2.55 | 5.00 ± 2.37 | 0.98 | 0.001 |
| Family members’ fear of COVID-19^c^ | 5.41 ± 2.37 | 5.53 ± 2.07 | 0.23 | 0.05 |
| Family well-being |  |  |  |  |
| Family health^c^ | 6.16 ± 2.09 | 6.49 ± 1.95 | <0.001 | 0.16 |
| Family harmony^c^ | 6.43 ± 2.17 | 6.76 ± 1.98 | <0.001 | 0.16 |
| Family happiness^c^ | 6.18 ± 2.12 | 6.51 ± 1.93 | <0.001 | 0.17 |
| Psychological distress |  |  |  |  |
| Anxiety symptoms^d^ | 3.75 ± 1.63 | 3.71 ± 1.47 | 0.53 | 0.03 |
| Depressive symptoms^d^ | 3.61 ± 1.57 | 3.50 ± 1.47 | 0.10 | 0.08 |

^a^ Monthly household income per person were grouped as “lower” or “higher” with reference to the size-specific median monthly household income in Hong Kong’s census statistics.

^b^ Effect size: Cohen’s *d* for the continuous variable: 0.20–0.50, small; 0.50–0.80, medium; ≥ 0.80, large.

^c^ A single item ranges from 0 to 10, with higher scores indicating higher levels.

^d^ The subscale of four-item Patient Health Questionnaire measuring anxiety/depressive symptoms, ranging from 0 to 6 with higher scores indicating higher anxiety/depressive symptoms.


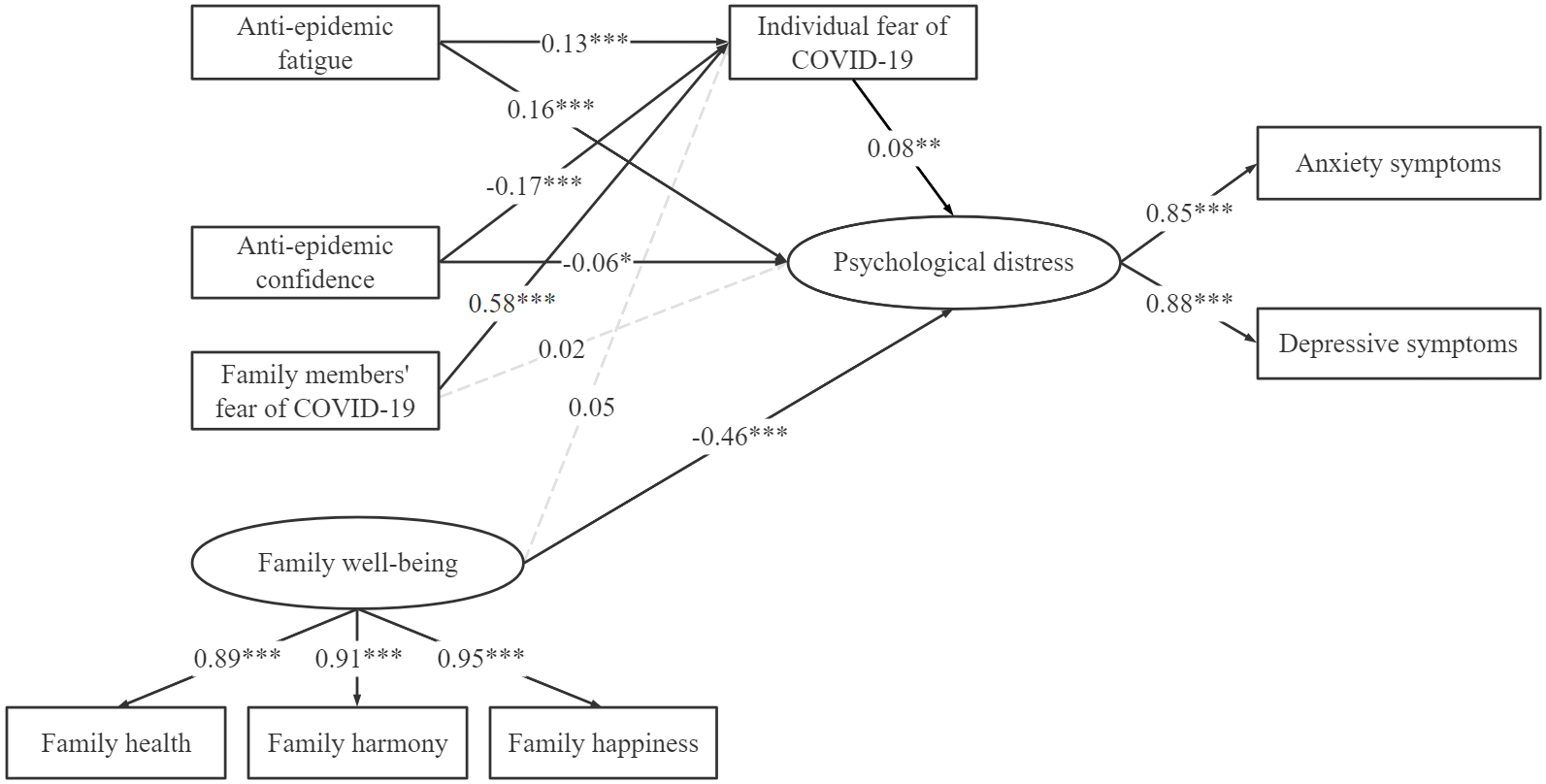


**Supplementary Figure 1.** Path diagram of the structural equation model

All path coefficients are standardized. Solid lines indicate significant associations. Dotted lines indicate non-significant associations. Error terms are not shown in the figure. Anti-epidemic fatigue/anti-epidemic confidence/individual fear of COVID-19/family members’ fear of COVID-19 are indicated by a single item ranging from 0 to 10, with higher scores indicating higher levels. Family well-being is a latent variable indicated by family health, family harmony, and family happiness (each ranging from 0 to 10, with higher scores indicating higher levels). Psychological distress is a latent variable indicated by anxiety and depressive symptoms (each ranging from 0 to 6, with higher scores indicating higher levels). This model was adjusted by sex, age group, education level, monthly household income per person, and number of cohabitants. **P* < 0.05, ***P* < 0.01, ****P* < 0.001.


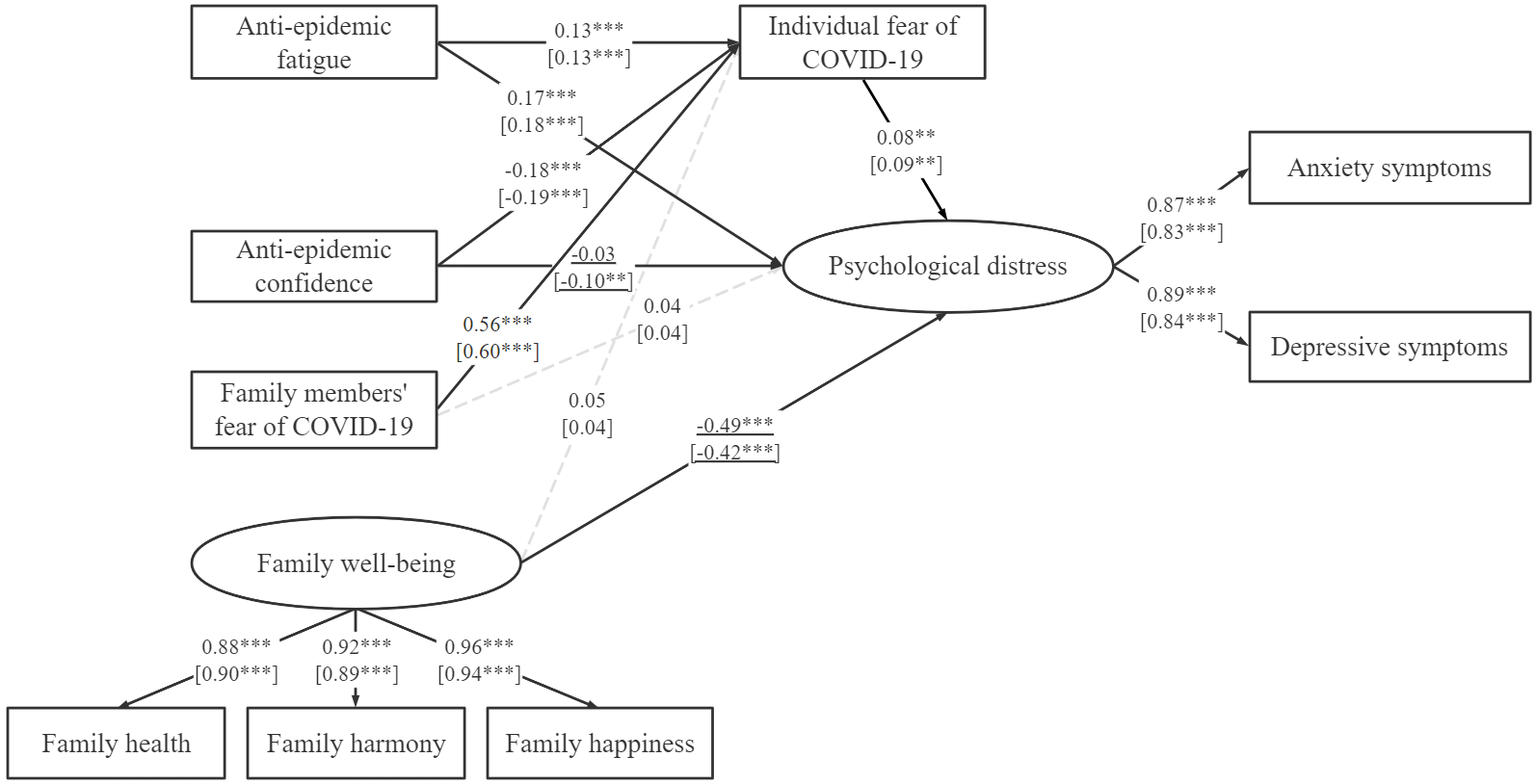


**Supplementary Figure 2.** Results of partially constrained structural equation models stratified by monthly household income per person

Path coefficients without the square brackets are for the group of lower monthly household income per person, and path coefficients with the square brackets are for the group of higher monthly household income per person (compared to the median level in Hong Kong’s census statistics). Coefficients with underlines indicate that there was a statistically significant difference in the path coefficients between lower and higher income groups. All path coefficients are standardized. Solid lines indicate significant associations. Dotted lines indicate non-significant associations. Error terms are not shown in the figure. Anti-epidemic fatigue/anti-epidemic confidence/individual fear of COVID-19/family members’ fear of COVID-19 are indicated by a single item ranging from 0 to 10, with higher scores indicating higher levels. Family well-being is a latent variable indicated by family health, family harmony, and family happiness (each ranging from 0 to 10, with higher scores indicating higher levels). Psychological distress is a latent variable indicated by anxiety and depressive symptoms (each ranging from 0 to 6, with higher scores indicating higher levels). This model was adjusted by sex, age group, education level, monthly household income per person, and number of cohabitants. **P* < 0.05, ***P* < 0.01, ****P* < 0.001.
